# Combination Design of Time-Dependent Magnetic Field and Magnetic Nanocomposites to Guide Cell Behavior

**DOI:** 10.3390/nano10030577

**Published:** 2020-03-22

**Authors:** Teresa Russo, Valentina Peluso, Antonio Gloria, Olimpia Oliviero, Laura Rinaldi, Giovanni Improta, Roberto De Santis, Vincenzo D’Antò

**Affiliations:** 1 Institute of Polymers, Composites and Biomaterials, National Research Council of Italy, V.le J.F. Kennedy 54-Mostra d’Oltremare Pad. 20, 80125 Naples, Italy; teresa.russo@unina.it (T.R.); rosantis@unina.it (R.D.S.); 2 Department of Neurosciences, Reproductive and Odontostomatological Sciences, University of Naples Federico II, 80131 Naples, Italy; valentina.peluso2@unina.it (V.P.); olivier@unina.it (O.O.); vincenzo.danto@unina.it (V.D.); 3 Department of Molecular Medicine and Medical Biotechnologies, University of Naples Federico II, 80131 Naples, Italy; laurarinaldi2000@yahoo.it; 4 Department of Public Health, University of Naples Federico II, 80131 Naples, Italy; ing.improta@gmail.com

**Keywords:** design of magnetic nanocomposite substrates, magnetic stimulation, reverse engineering/image analysis, mechanical properties and cell–material interaction, bone tissue engineering

## Abstract

The concept of magnetic guidance is still challenging and has opened a wide range of perspectives in the field of tissue engineering. In this context, magnetic nanocomposites consisting of a poly(ε-caprolactone) (PCL) matrix and iron oxide (Fe_3_O_4_) nanoparticles were designed and manufactured for bone tissue engineering. The mechanical properties of PCL/Fe_3_O_4_ (80/20 w/w) nanocomposites were first assessed through small punch tests. The inclusion of Fe_3_O_4_ nanoparticles improved the punching properties as the values of peak load were higher than those obtained for the neat PCL without significantly affecting the work to failure. The effect of a time-dependent magnetic field on the adhesion, proliferation, and differentiation of human mesenchymal stem cells (hMSCs) was analyzed. The Alamar Blue assay, confocal laser scanning microscopy, and image analysis (i.e., shape factor) provided information on cell adhesion and viability over time, whereas the normalized alkaline phosphatase activity (ALP/DNA) demonstrated that the combination of a time-dependent field with magnetic nanocomposites (PCL/Fe_3_O_4_ Mag) influenced cell differentiation. Furthermore, in terms of extracellular signal-regulated kinase (ERK)1/2 phosphorylation, an insight into the role of the magnetic stimulation was reported, also demonstrating a strong effect due the combination of the magnetic field with PCL/Fe_3_O_4_ nanocomposites (PCL/Fe_3_O_4_ Mag).

## 1. Introduction

Tissue engineering and stem cell-based therapies are the most challenging fields in regenerative medicine. Tissue engineering is based on the synergistic combination of cells and appropriate scaffolds acting as functionally supportive biomolecules. A common scaffold is an interconnected porous structure that supports cell adhesion, proliferation and differentiation while promoting the extracellular matrix (ECM) analogue deposition that is necessary for tissue regeneration.

Natural (e.g., alginate, collagen, chitosan, agarose, hyaluronic acid, and fibrin) and synthetic polymers (e.g., poly(ε-caprolactone)) have been employed to fabricate scaffolds for tissue engineering [1,2]. In this context, mechanical features are as important as hard tissues (e.g., bone), and they are stiffer (higher elastic modulus) and stronger (higher strength) compared to soft tissues [3,4,5,6,7].

If compared to other aliphatic polyesters, poly(ε-caprolactone) (PCL) has better viscoelastic and rheological properties, also resulting in it being easily workable and manipulatable [8]. Anyway, its mechanical properties are not suitable for high load-bearing applications (e.g., bone) [8,9]. Moreover, PCL hydrophobicity tends to inhibit cell adhesion and proliferation [9]. 

In regard to bone tissue engineering, different osteoinductive and osteogenic inorganic fillers (e.g., hydroxyapatite and bioactive glasses) are generally employed to enhance the biological and mechanical performances of PCL-based scaffolds [9]. 

Composite structures consisting of PCL reinforced with tricalcium phosphate (TCP) [8] or electrospun PCL nanofiber scaffolds with different amount of graphene oxide and graphene oxide surface grafted with poly(ethylene glycol) have been developed [9,10].

The inclusion of nanofillers in the PCL matrix up to a threshold concentration usually improves its mechanical properties [9].

A recent study also focused on the development of PCL/layered double hydroxide (LDH) microsphere-aggregated nanocomposite scaffolds as suitable candidates for bone tissue engineering [11]. The results demonstrated that the inclusion of LDH nanoparticles improved the osteogenic differentiation and mechanical properties (e.g., compressive modulus) of mesenchymal stem cells. depending upon the LDH amount [11].

The grafting of carboxymethyl chitosan on electrospun PCL nanofibers was also proposed to manufacture scaffolds for bone tissue regeneration. The synergic effect of external stimulation factors (e.g., β-carotene and electromagnetic field) on the osteodifferentiation of adipose mesenchymal stem cells was analyzed [12].

Differently from conventional composites, nanocomposites based on a polymer matrix and inorganic-reinforcing nanofillers seem to better reproduce the natural structure of bone, a natural nanocomposite, and would represent a relevant candidate for bone tissue engineering. In addition, it has been widely demonstrated that nanocomposites induce a more efficient cell response and generally possess improved mechanical performance [1,7].

The use of magnetic nanoparticles in combination with an external magnetic field may represent an intriguing strategy to enhance bone tissue regeneration, as it has been reported to influence cellular metabolism. In the field of medicine, static and pulsed magnetic fields have been commonly used as components of the magnetic resonance technique, as well as to increase wound healing and to improve bone regeneration. Furthermore, recent data have also shown the mechanism of how a static magnetic field may influence biochemical properties using stem cells and different cell populations. 

The findings obtained to date suggest the potential use of therapies based on magnetic fields due to their easy application and possible effects on cells and organisms [13,14,15,16,17,18,19]. The concept of magnetic guidance has been extensively studied in the biomedical field (e.g., drug delivery, hyperthermia treatment for cancer, magnetic cell-seeding procedures, the stimulation of cell constructs, and the control of cell proliferation and differentiation) [19,20,21,22,23,24,25,26,27,28,29,30,31,32].

In this scenario, magnetic nanoparticles (MNPs) provide attractive possibilities as a direct consequence of their peculiar physical properties and their sizes. The application of an external magnetic field may also allow for the manipulation of their magnetic features, as well as the immobilization and/or transportation of the MNPs themselves and magnetic bioaggregates [33,34]. 

The potential to magnetically switch-on/switch-off a nanocomposite scaffold, consisting of a polymer matrix loaded with MNPs, could also be considered to deliver biomolecules (e.g., angiogenic factors) and stem cells, as well as to enhance cell adhesion, proliferation, and differentiation [17]. Even if many concerns remain about the long-term effects of iron-oxide-based phases such as maghemite (γ-Fe_2_O_3_) and magnetite (Fe_3_O_4_) [34,35,36,37] in the human body, over the past few years, the idea to design a fixed “station” whose magnetization can be switched on or off through the application or removal of external magnetic fields [14], respectively, has clearly led to the development of magnetic nanocomposite structures consisting of poly(ε-caprolactone) (PCL) loaded with Fe_3_O_4_ or iron-doped hydroxyapatite (FeHA) nanoparticles, as well as a challenging and programmed biofactor release [33,34,38]. 

Basically, the distribution of magnetic flux distribution can be altered by the presence of a magnetic scaffold, and magnetic field lines can be much more concentrated near and inside the scaffold. Magnetic gradients can be generated to allow the scaffold to attract and take up cells or bioagents bound to MNPs, which should function as transportation shuttles towards the magnetic scaffold [14]. 

The magnetic performances of both PCL/Fe_3_O_4_ and PCL/FeHA nanocomposites have already been analyzed, and they showed a sigmoidal shape of the magnetization curve and a very low coercive field at 37 °C [34,38].

On the other hand, magnetic force could lead to changes in microenvironments, cell membranes, matrixes, cytoskeletons, and nucleoproteins. Therefore, signals can be transduced to the cell nucleus, modulating and promoting many biological responses. Several signaling pathways, such as the mitogen-activated protein kinase (MAPK) pathway, are generally involved [39,40,41]. The expression of MAP protein kinases plays a fundamental regulatory role in cellular biology [41,42]. The phosphorylation of several substrate proteins, involving transcription factors, protein kinases and phosphatases, and further proteins, can be catalyzed by the activated MAP kinases. 

The MAPK pathway consists of a series of Ser/Thr kinases, including extracellular signal-regulated kinase (ERK)1/2, c-Jun N-terminal kinase (JNK), p38, and ERK5 families, allowing for the regulation of the activity of specific transcription factors after the phosphorylation cascade. 

In regard to bone, tissue resorption and formation are related to the MAPK signaling pathway. To date, the effect of magnetic fields on osteogenic marker expression through different signaling pathways, including the protein kinase A (PKA) and MAPK, remains unclear [43,44,45]. 

Accordingly, the aim of the current research was to provide further insight into the combination design of a time-dependent magnetic field and PCL/Fe_3_O_4_ (80/20 w/w) nanocomposites for bone tissue engineering. The mechanical properties of PCL/Fe_3_O_4_ nanocomposites were first evaluated through small punch tests, whereas the combined effect of the magnetic stimulation and PCL/Fe_3_O_4_ nanocomposites on the behavior of human mesenchymal stem cells (hMSCs) was analyzed, with a focus on the MAPK signaling pathway. 

## 2. Materials and Methods 

### 2.1. Design and Manufacturing of PCL/Fe_3_O_4_ Nanocomposite Substrates

Nanocomposite pellets were first prepared according to a procedure already reported for the solution preparation and MNP dispersion [34,38,46]. Tetrahydrofuran (THF, Sigma-Aldrich, St. Louis, MO, USA) was used to dissolve poly(ε-caprolactone) (weight-average molecular weight M_w_ = 65,000, Aldrich, St. Louis, MO) pellets at room temperature. Polyvinylpyrrolidone (PVP)-coated Fe_3_O_4_ (99.5%, 25 nm, 0.2 wt % PVP, NanoAmor, Houston, TX) nanoparticles and, successively, ethanol were added to the polymer solution. A polymer/filler (PCL/Fe_3_O_4_) weight ratio (w/w) of 80/20 was employed. In regard to the nanoparticle dispersion, an ultrasonic bath (Branson 1510 MT, Danbury, CT) was also utilized. 

PCL/Fe_3_O_4_ (80/20 w/w) nanocomposite pellets were prepared from the obtained homogeneous paste after removing the solvent. The pellets were processed via a melting and molding technique to manufacture PCL and PCL/Fe_3_O_4_ (80/20 w/w) substrates. In brief, PCL or PCL/Fe_3_O_4_ pellets were heated to 100 °C, and the material was subsequently poured into a Teflon mold, allowing it to cool. 

A mylar strip was also placed on the top of the mold, and an appropriate load was distributed to achieve a flat surface for the specimens.

In particular, disk-shaped specimens (diameter and thickness of 6.4 and 0.5 mm, respectively) were manufactured to perform small punch tests, whereas specimens with a larger diameter (10 mm) were employed for biological analyses.

### 2.2. Small Punch Test

Small punch tests were performed on PCL and PCL/Fe_3_O_4_ disk-shaped specimens (diameter of 6.4 and thickness of 0.5 mm) according to the ASTM F2183. Using a hemispherical head punch, disk-shaped specimens were loaded axisymmetrically in bending at a constant displacement rate of 0.5 mm/min until failure occurred. Load and displacement values were recorded during the test. 

The tests were carried out with an INSTRON 5566 testing machine (Norwood, MA, USA).

### 2.3. Scanning Electron Microscopy 

The surface morphologies of the PCL and PCL/Fe_3_O_4_ substrates were analyzed with scanning electron microscopy (SEM) (FEI Quanta 200 FEG apparatus, The Netherlands).

### 2.4. Cell Culture

Human mesenchymal stem cells (hMSCs, Millipore, Germany), at the fourth passage, were cultured in Dulbecco’s modified eagle medium (DMEM, Microtech, Italy) supplemented with 10% (v/v) fetal bovine serum (FBS, Gibco, Thermo Fisher Scientific, Waltham, MA, USA), 2 mM glutamine, and antibiotics (penicillin G sodium 100 U/mL, streptomycin 100 g/mL) at 37 °C and 5% CO_2_.

PCL and Fe_3_O_4_ substrates were prepared by soaking the structures in a solution of ethanol and antibiotics (penicillin/streptomycin), washed in phosphate-buffered saline (PBS, Sigma-Aldrich, Milan, Italy), and pre-wetted in FBS. hMSCs were seeded onto the substrates using a density of 1.0 x 10⁴ cells/sample. After seeding, the cell-laden structures were incubated for 2 h (37 °C, 5% CO_2_) and, successively, 1.5 mL of culture medium was added to each well in a 48-well plate.

#### 2.4.1. Magnetic Stimulation

A time-dependent magnetic stimulation of the cell-laden constructs were performed at 1 day after cell seeding. Time-dependent means that the magnetic field varies as time (t) increases, and a sinusoidal wave is generally considered one of the commonly employed representation.

**Figure 1:**
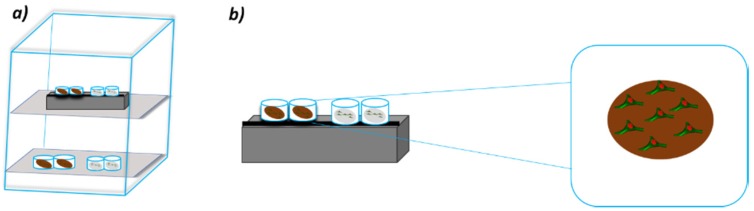
A scheme of the experimental setup that was employed for magnetic stimulation. (**a**) Incubator equipped with electromagnet; (**b**) further details related to cell-laden constructs and stimulation system.

Specifically, an external sinusoidal magnetic field was discontinuously applied for 6 h per day (20 intervals at 18 min each) benefiting from an optimized procedure [46]. Further cell-laden substrates were used as controls because they were placed in the same conditions without magnetic stimulation. A scheme of the experimental setup is shown in Figure 1. 

The cell-laden constructs were exposed to a time-dependent magnetic field with an electromagnet placed below the wells. 

In order to avoid any kind of mutual influence, adequate technical solutions were adopted for the adjacent cell culture wells [46]. 

The test was performed three times in triplicate. As generally reported, the magnetically-stimulated PCL and PCL/Fe_3_O_4_ substrates were marked as PCL Mag and PCL/Fe_3_O_4_Mag, respectively, where “Mag” was used to indicate the application of the magnetic field.

#### 2.4.2. Cell Metabolic Activity 

The Alamar Blue assay (AbD Serotec Ltd., UK) was used to assess cell viability and proliferation. At 1, 4, 7, 14, and 21 days after cell seeding, the cell-laden substrates were rinsed with PBS (Sigma–Aldrich, Italy) and 200 μL of DMEM without phenol red (HyClone, UK) containing 10% (v/v) Alamar Blue was added for each sample. The samples were incubated in 5% CO_2_ diluted atmosphere for 4 h at 37 °C. 

After removing one hundred microliters of the solution, it was transferred to the well plate. A spectrophotometer (Sunrise, Tecan, Männedorf, Zurich, Switzerland) was utilized, and the optical density was measured at wavelengths of 570 and 595 nm.

The experiments were done at least three times in triplicate.

#### 2.4.3. Alkaline Phosphatase Activity 

Samples were removed from the medium and washed twice with PBS at days 3, 7, and 14. The cell-laden substrates were then incubated in 1 mL of a lysis buffer and centrifugated. A cell density of 1 × 10⁴ cells/sample was employed. 

The alkaline phosphatase (ALP) activity was measured with an enzymatic assay (SensoLyte pNPP alkaline phosphatase assay kit - AnaSpec Inc., Fremont, CA, USA), which was based on the p-nitrophenyl phosphate (pNPP). Normalized ALP activity (ALP/DNA) was calculated by dividing the ALP activity over the DNA content with the Quant-iT PicoGreen assay kit (Molecular Probes Inc., Eugene, OR, USA), which allows for the detection and quantification of DNA. 

According to the manufacturer’s protocol, the working solutions were prepared and the procedure was followed.

#### 2.4.4. Confocal Laser Scanning Microscopy

Confocal laser scanning microscopy (CLSM) was also employed as an optical imaging technique for the analysis of cell adhesion and spreading at 4, 7, and 14 days after seeding, using a Zeiss LSM 510/ConfoCor 2 system (Oberkochen, Germany) equipped with argon and helium–neon lasers and with a 10X objective. In brief, rhodamine phalloidin staining was performed, constructs were imaged, and actin filaments were visualized. 

CLSM images were successively analyzed with Image J software (NIH, Bethesda, MD, USA) to determine the cell morphology [2,46,47].

The cell shape factor (*Φ*) was calculated as follows:

(1) $$\Phi =\frac{4\pi A}{P^{2}}$$

where *A* and *P* represent the area and the perimeter of a cell, respectively. 

The greatest area-to-perimeter ratio is obtained for circular objects, and a perfect circle has a shape factor of 1. Conversely, a thin thread-like object shows the lowest shape factor that approaches zero [2,46,47].

#### 2.4.5. Immunoblot Analysis 

The following primary antibodies were used: rabbit phospho ERK (Thr202/Tyr204) (Cell Signaling) and rabbit ERK1/2 (Santa Cruz). Polyclonal antibodies were employed at working dilutions of 1:1000. Antibody protein complexes were detected by horseradish peroxidase (HRP)-conjugated antibodies and an enhanced chemiluminescent (ECL) system (both from Amersham Pharmacia, Piscataway Township, NJ, USA).

PCL and PCL/Fe_3_O_4_ substrates were first prepared by soaking the structures in a solution of ethanol and antibiotics (penicillin/streptomycin), washed in PBS (Sigma-Aldrich, Italy) and pre-wetted in FBS. hMSCs (density of 1.8 × 10^5^ cells/sample) were seeded onto the substrates (diameter and thickness of 34 and 0.5 mm, respectively) in a 6-well plate. 

After seeding, the cell-laden structures were incubated for 2 h (37 °C, 5% CO₂) and, successively, 1.5 mL of a culture medium was added to each well. 

Four days after magnetic stimulation (6 h per day for 20 intervals of 18 min each), cell-laden substrates were serum-deprived overnight and left untreated or treated with epidermal growth factor (EGF—100 ng/mL; treatment times of 15 and 30 min). 

The cells were collected with trypsin and then centrifuged, and the obtained pellets were stored at -80° C. hMSCs were lysed in a saline buffer containing 1 mM ethylenediaminetetraacetic acid (EDTA), 50 mM Tris-HCl (pH 7.5), 70 mM NaCl, and 1% Triton. The lysates were cleared by centrifugation at 15,000 x g for 10 min and quantified by the Bradford method. Sodium dodecyl sulfate polyacrylamide gel electrophoresis (SDS-PAGE) was carried out on cell lysates, and the proteins were transferred to a nitrocellulose membrane for 3 h. Filters were blocked for 1 h at room temperature in Tween-20 Tris-buffered saline (TTBS) (TBS-Sigma, 0.1% Tween 20, pH 7.4) with 5% w/v non-fat dry milk. 

Blots were then incubated with a primary antibody (p-ERK; ERK1/2) overnight. They were washed three times with a TTBS buffer and incubated for 1 h with a secondary antibody (peroxidase-coupled anti-rabbit) in TTBS-5% (w/v) non-fat dry milk. Reactive signals were detected by an ECL Western blotting analysis system. The autoradiography images were acquired and analyzed with Image-J software (NIH, Bethesda, MD, USA).

Thus, cell-laden substrates were treated with EGF at different time points (15 and 30 min) in the absence or presence of a time-dependent magnetic field applied for 6 h per day (20 intervals of 18 min each) for 4 days. 

The magnetically-stimulated cell-laden substrates were compared to the control groups (unstimulated polymeric and nanocomposite substrates) in the presence and absence of EGF.

### 2.5. Statistical Analysis 

Data are reported as mean value ± standard deviation and analyzed by ANOVA followed by Bonferroni post hoc test. A value of *p* < 0.05 was considered statistically significant.

## 3. Results

### 3.1. Small Punch Test

Load-displacement curves from small punch tests on disk-shaped specimens (PCL and PCL/Fe_3_O_4_) displayed an initial linear trend, a subsequent decreasing slope, a maximum load, and, then, a final decrease of the load until failure occurred.

The initial local maximum (peak load) and the area under the load-displacement curve (work to failure) were assessed (Table 1).

PCL/Fe_3_O_4_ substrates provided values of peak load that were significantly higher than those achieved for PCL (*p* < 0.05). In terms of work to failure, no statistically significant differences were found between the two groups (*p* > 0.05). 

### 3.2. Scanning Electron Microscopy

**Figure 2:**
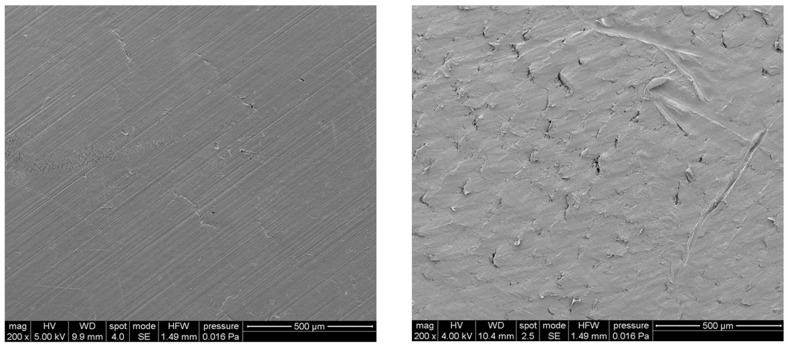
Typical SEM images of poly(ε-caprolactone) (PCL) (left) and PCL/Fe_3_O_4_ (right) substrates. Scale bar: 500 μm.

SEM images of polymeric (PCL) and nanocomposite (PCL/Fe_3_O_4_ 80/20 w/w) substrates are reported in Figure 2. The surface topography of the nanocomposites was clearly influenced by the presence of aggregates and MNPs distributed in the matrix.

### 3.3. Cell Metabolic Activity

**Figure 3:**
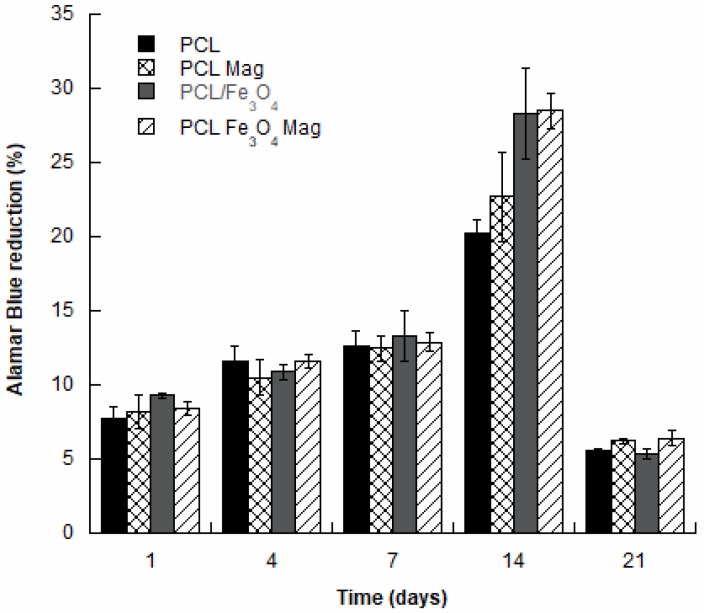
Percentage of Alamar Blue reduction evaluated for PCL, PCL/Fe_3_O_4_, PCL Mag and PCL/Fe_3_O_4_ Mag at different time points.

Cell viability and proliferation were analyzed for the different kinds of substrates, and the results are reported as a percentage of Alamar Blue reduction (Figure 3). 

The results evidenced that all samples supported the adhesion and proliferation of hMSCs. As reported, the number of viable cells was associated with the magnitude of dye reduction. 

A significant increase (*p* < 0.05) of Alamar Blue reduction was evident over time, suggesting that hMSCs can survive and proliferate. One day after seeding, the results indicated that hMSCs were viable on both PCL and PCL/Fe_3_O_4_ substrates, with and without magnetic stimulation. An increase in the value of the percentage of Alamar Blue reduction was evident up to 14 days after seeding. Even though no statistically significant differences (*p* > 0.05) were observed among the different groups at 1, 4, and 7 days, at day 14, the percentage of Alamar Blue reduction was significantly higher (*p* < 0.05) for PCL/Fe_3_O_4_ and PCL/Fe_3_O_4_ Mag if compared to the PCL substrates. 

However, no statistically significant differences were found between the PCL/Fe_3_O_4_ and PCL/Fe_3_O_4_ Mag substrates (*p* > 0.05). 

In addition, at 21 days after cell seeding, a significant decrease (*p* < 0.05) in the percentage of Alamar Blue reduction was found for the different kinds of cell-laden constructs (Figure 3). 

### 3.4. Alkaline Phosphatase Activity

The alkaline phosphatase activity was measured at 3, 7, and 14 days for each group (PCL, PCL/Fe_3_O_4_, PCL Mag, and PCL/Fe_3_O_4_ Mag) and normalized to DNA content (ALP/DNA) to quantitatively assess early osteogenic differentiation. 

**Figure 4:**
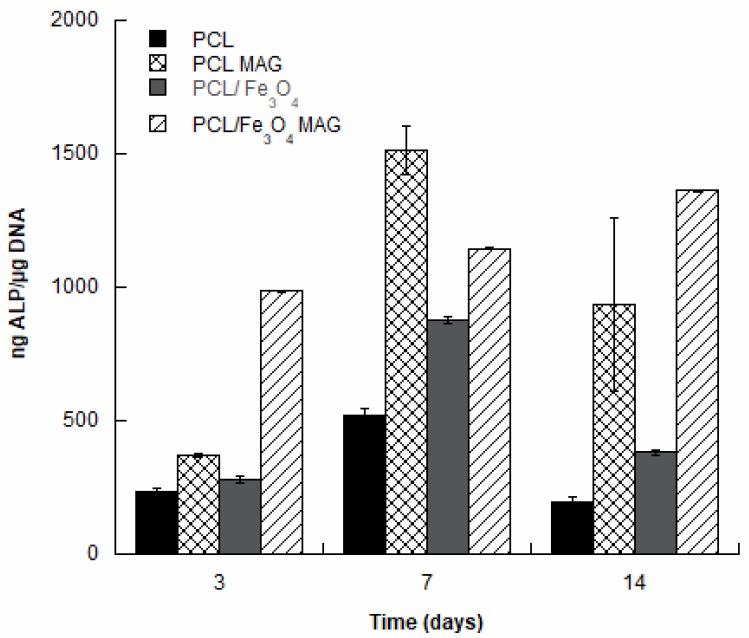
Normalized alkaline phosphatase (ALP) activity (ALP/DNA) for PCL, PCL/Fe_3_O_4_, PCL Mag, and PCL/Fe_3_O_4_ Mag at 3, 7, and 14 days after cell seeding.

Figure 4 reports some differences in terms of results, as the ALP/DNA ratio peaked at 7 days in the case of PCL, PCL Mag and PCL/Fe_3_O_4_, whereas the values significantly increased (*p* < 0.05) over the analyzed time period (from 3 to 14 days) for PCL/Fe_3_O_4_ Mag. 

In the case of both PCL Mag and PCL/Fe_3_O_4_, significantly higher values were found in comparison to PCL (*p* < 0.05). 

However, at different time points, the unstimulated PCL/Fe_3_O_4_ showed a lower ALP activity compared to the magnetically-stimulated PCL (PCL Mag). The observed differences were statistically significant (*p* < 0.05). 

On the other hand, the effect of the magnetic stimulation on PCL/Fe_3_O_4_ led to the highest ALP activity at 3 days, as well as an increasing trend over time (see PCL/Fe_3_O_4_ Mag).

### 3.5. Confocal Laser Scanning Microscopy

CLSM performed on all the cell-laden substrates provided qualitative results in terms of cell adhesion and spreading at 4, 7, and 14 days after seeding. 

**Figure 5:**
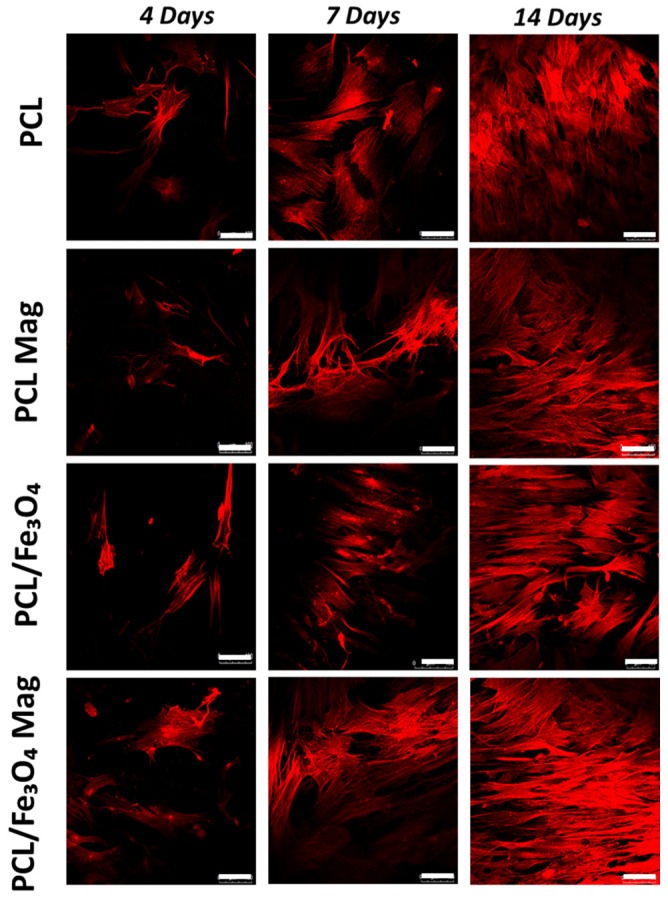
Confocal laser scanning microscopy (CLSM) analysis on cell-laden substrates at different time points. Images of rhodamine phalloidin-stained actin filaments (red). (left column) From top to bottom: PCL, PCL Mag, PCL/Fe_3_O_4_, and PCL/Fe_3_O_4_ Mag at 4 days. (middle column) From top to bottom: PCL, PCL Mag, PCL/Fe_3_O_4_ 80/20, and PCL/Fe_3_O_4_ 80/20 Mag at 7 days. (right column) From top to bottom: PCL, PCL Mag, PCL/Fe_3_O_4_ 80/20, and PCL/Fe_3_O_4_ 80/20 Mag at 14 days. Scale bar: 100 µm.

The CLSM images confirmed the results obtained from the Alamar Blue assay. The number of viable cells on the nanocomposite substrates under magnetic stimulation strongly increased over time. Cell morphology also varied over time, changing from a geometry characterized by few ramifications to a thread-like geometry with an increased number of ramifications (Figure 5), thus suggesting the establishment of a higher number of cell–cell and cell–material interactions. 

**Figure 6:**
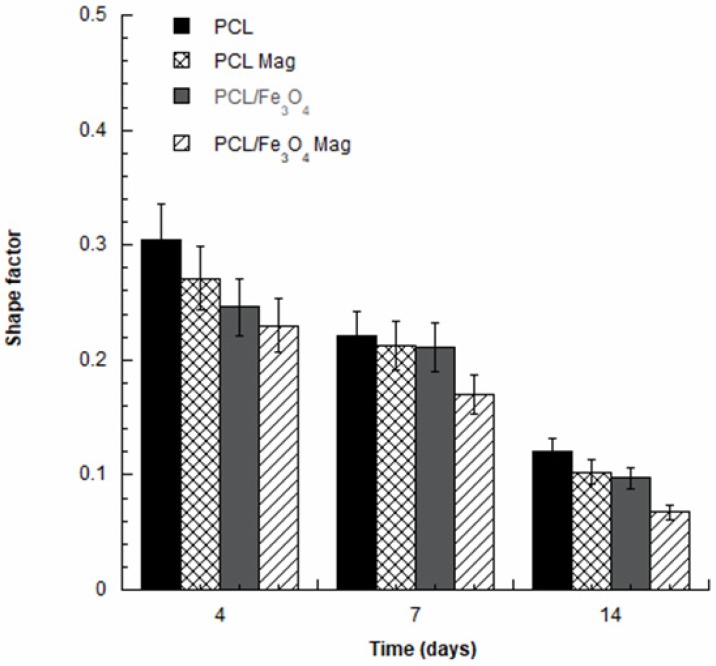
Shape factor evaluated from the CLSM images of human mesenchymal stem cells (hMSCs) on PCL and PCL/Fe_3_O_4_ substrates (with or without magnetic stimulation) at different time points.

Further studies of cell adhesion and spreading were carried out based on CLSM images with the aim of determining the shape factor of the cells. Figure 6 reports the shape factor at 4, 7, and 14 days.

As an example, in the case of PCL/Fe_3_O_4_ nanocomposites under magnetic stimulation (PCL/Fe_3_O_4_ Mag), the values of the cell shape factor significantly decreased (*p* < 0.05) from 0.23 ± 0.02 at day 4 to 0.06 ± 0.01 at day 14. 

Even though no statistically significant differences (*p* > 0.05) were observed among the several groups in terms of cell shape factor at day 4, significantly lower values (*p* < 0.05) were found in the case of PCL/Fe_3_O_4_ Mag at 7 and 14 days. Furthermore, no significant differences (*p* > 0.05) were found among PCL, PCL Mag, and PCL/Fe_3_O_4_ at 7 and 14 days.

### 3.6. Immunoblot Analysis

**Figure 7:**
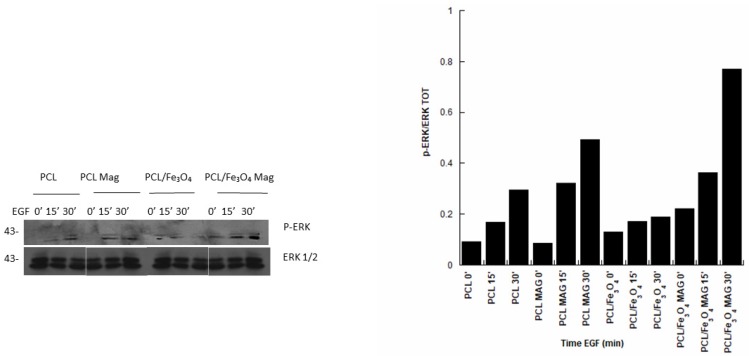
Effect of magnetic field on extracellular signal-regulated kinase (ERK)1/2 phosphorylation. Western blotting analyses were performed on substrates stimulated with a time-dependent magnetic field or left unstimulated. The experimental groups were treated with epidermal growth factor (EGF) at indicated time points (15 and 30 min). Cell-laden substrates were then lysed and subjected to immunoblot analysis with the indicated antibodies. PCL 0’, PCL/Fe_3_O_4_ 0’, PCL Mag 0’, and PCL/Fe_3_O_4_ Mag 0’ indicate cell constructs in the absence of EGF.

The p-ERK1/2 expression was investigated for the different kinds of cell-laden substrates. Specifically, the phosphorylation levels of ERK1/2 were monitored by Western blotting analysis. In regard to both PCL and PCL/Fe_3_O_4_, the presence of EGF led to phosphorylation levels of ERK1/2 that were generally higher than those obtained in the absence of EGF, also increasing with the EGF treatment time (Figure 7). Moreover, the magnetic stimulation further improved the phosphorylation levels of ERK1/2, also providing interesting results in the absence of EGF. 

However, the highest levels were found for PCL/Fe_3_O_4_ Mag (Figure 7). 

In Figure 7, samples marked as PCL 0’, PCL/Fe_3_O_4_ 0’, PCL Mag 0’, and PCL/Fe_3_O_4_ Mag 0’ represent cell constructs in the absence of EGF.

## 4. Discussion

The concept of magnetic guidance may be properly considered to design devices where many features related to the magneto-mechanical activation/stimulation of cell-laden constructs, magnetic cell-seeding techniques, and tailored cell proliferation and differentiation—as well as to the release of biomolecules or bioactive factors that can be linked to magnetic nanocarriers—can be simultaneously integrated [14,15,16,17,18,19,20,21,22,23,24,25,26,27,28,29,30,31,32,38,47,48].

The possibility to design magnetic nanocomposite substrates represents a great challenge in the field of bone tissue regeneration. The rationale should be the development of magnetic nanocomposite structures that may be magnetized in situ by applying an external magnetic field with the aim to control specific cellular processes. 

In regard to the preparation of polymer-based nanocomposites, over the past few years, ultrasonication has been considered an efficient methodology to enhance the dispersion of nanofillers in a polymer solution, allowing for the excitation of the resonance vibrations of nanofiller clusters and/or to break up them [49]. 

Further works on the development of PCL/MNP scaffolds have frequently reported the use of ultrasonication as a strategy to homogeneously disperse MNPs (e.g., magnetite and iron oxide-based nanoparticles) in different concentrations with respect to the total PCL mass [19,50].

In the current research, a procedure that had already been reported for the preparation of the PCL solution and MNPs (e.g., PVP-coated Fe_3_O_4_ and iron-doped hydroxyapatite) dispersion under ultrasonication [34,38,46] was adopted to prepare PCL/Fe_3_O_4_ pellets for the manufacturing of nanocomposites. Moreover, many studies have dealt with the high dispersion stability of PVP-coated iron-oxide nanoparticles in different solvents, sonochemical methods, andsonication methods in the presence of PVP as stabilizer to produce well-dispersed nanoparticles [51,52].

As frequently reported, in polymer nanocomposites, the different ductility values between the inorganic nanofillers and the polymeric matrix generally cause stress concentration and discontinuities in the stress transfer mechanism at the nanoparticle/matrix interface [46,48]. 

According to previously reported data, a decrease of the functional properties may be observed beyond a threshold concentration of nanoparticles, as they can act as “weak points” instead of a reinforcement for the polymeric matrix, thus weakening structures [46,48,53]. 

Additionally, magnetization measurements had already been performed on PCL/Fe_3_O_4_ nanocomposites, and the results provided information in terms of a very low coercive field and saturation magnetization levels [34]. As for some physical, magnetic, and functional characteristics of the employed PCL/Fe_3_O_4_ nanoparticles (99.5%, 25 nm, 0.2 wt % PVP, NanoAmor, Houston, TX), previous studies [34,54] on the development of additively manufactured PCL/Fe_3_O_4_ scaffolds have demonstrated that the inclusion of these MNPs led to magnetization curves with saturation magnetization values ranging from 4 to 6 emu/g, depending upon the amount of MNPs. Starting from magnetization curves for the PCL/Fe_3_O_4_ scaffolds and fitting of the experimental data with the Langevin function, a particle diameter of 28 nm was estimated [34].

In this scenario, the current investigation provided a further insight into the combination design of a time-dependent magnetic field and PCL/Fe_3_O_4_ (80/20 w/w) nanocomposites to potentially guide cell behavior.

The mechanical properties of PCL/Fe_3_O_4_ nanocomposites developed using melting and molding techniques were evaluated through the small punch test, which is a reproducible miniature specimen test method. 

Correlations between mechanical properties that were evaluated through uniaxial tensile tests and small punch tests were found for some low-alloy steels, providing analytical formulations that properly described the uniaxial stress–strain behavior [55]. 

This test method had already been employed for the evaluation of the mechanical properties of ultra-high molecular weight polyethylene employed in surgical implants and retrieved acrylic bone cements, as well as of PCL loaded with organic–inorganic hybrid fillers and PCL/iron-doped hydroxyapatite nanocomposite substrates [6,38]. 

It has been well documented that the data obtained from small punch tests do not provide information on yield stress and Young’s modulus, especially in the case of polymer-based nanocomposites, as the loading configuration leads to lateral bending and large biaxial deformations [56]. 

Taking into account a small deformation region and the initial slope of the obtained curves, even though finite element analysis can be performed to assess mechanical properties such as the Young’s modulus, this approach is sensitive to some parameters including the further mechanical characteristics of the specimen [56]. Accordingly, the addition of nanofillers influence the elastic and post-yield behavior and the neglecting of these features most often leads to an incorrect evaluation of the modulus. Thus, it would be complex to separately assess these properties using a single test configuration [56]. 

For this reason, in the current research, relative mechanical properties were reported as punching properties that were evaluated by small punch test while allowing for a comparison between the PCL and PCL/Fe_3_O_4_ substrates. The peak load and the work to failure were determined as a relative measure of the strength and the ability of the material to absorb energy before breaking (i.e., toughness), respectively. 

The inclusion of Fe_3_O_4_ nanoparticles improved the punching properties, as the values of peak load achieved for PCL/Fe_3_O_4_ nanocomposites was significantly higher (*p* < 0.05) than those obtained for PCL without negatively affecting the work to failure (*p* > 0.05) (Table 1), even if SEM images showed a surface topography where the presence of aggregates was evident. 

Though the results summarized in Table 1 (peak load and work to failure) do not yield functional information in terms of mechanical properties for bone tissue engineering applications, it is worth noting that previous works [34,54] on the design of additively manufactured PCL/Fe_3_O_4_ nanocomposite scaffolds have preliminarily demonstrated the potential to match the strength and modulus of the human cancellous bone (4–12 MPa and 0.02–0.5 GPa, respectively [47,57]) by tailoring composition, architectural features (e.g., lay-down pattern), pore shape and size.

However, Alamar Blue assay, normalized ALP activity (ALP/DNA), CLSM analysis, cell shape factor, and p-ERK1/2 expression provided interesting and, in many cases, unreported information, also suggesting how the synergistic combination of PCL/Fe_3_O_4_ nanocomposites with a discontinuous application of an external magnetic field (6 h per day with 20 intervals of 18 min each) may impact cell behavior.

The discontinuous magnetic field would seem to significantly influence the behavior of hMSCs in terms of cell viability and differentiation. 

Even though an improvement of cell viability should be probably ascribed to the effect of magnetic stimulation, surface topography, and chemistry (Figure 3), as higher values of the percentage reduction of Alamar Blue were found in the case of PCL/Fe_3_O_4_ and PCL/Fe_3_O_4_ Mag at day 14, the results in terms of normalized ALP activity evidenced important differences affecting the cell differentiation process (Figure 4). 

The effect of the material alone and in combination with the magnetic field was analyzed. In the absence of magnetic stimulation, the effect of surface topography and chemistry on cell osteogenic differentiation was clearly evident, as PCL/Fe_3_O_4_ nanocomposite showed a level of ALP activity that was higher than that found for PCL (Figure 4). 

For each material (PCL or PCL/Fe_3_O_4_), the application of a time-dependent magnetic field provided a higher level of ALP activity for the magnetically-stimulated substrates (PCL Mag or PCL/Fe_3_O_4_ Mag), if compared to the unstimulated ones (PCL or PCL/Fe_3_O_4_).

In addition, for each material (PCL or PCL/Fe_3_O_4_), the applied time-dependent magnetic field differently influenced the behavior of hMSCs over time, since the ALP/DNA ratio peaked at 7 days for PCL Mag, as with all the unstimulated substrates, whereas the magnetic stimulation led to increasing levels of ALP activity in the case of PCL/Fe_3_O_4_ (see PCL/Fe_3_O_4_ Mag in Figure 4), also resulting in a prolonged differentiation. 

The obtained results suggested that the combination of PCL/Fe_3_O_4_ nanocomposites with a time dependent magnetic field (PCL/Fe_3_O_4_ Mag) was able to provide and improve the long-term maintenance of hMSC differentiation, which was more rapidly lost in the case of both magnetically-stimulated PCL (PCL Mag) and unstimulated substrates (PCL and PCL/Fe_3_O_4_).

CLSM images and cell shape factor allowed us to further analyze cell adhesion and spreading at different time points, indicating a correlation between the reduction in cell shape factor over time with the establishment of multiple cellular extensions and, hence, an increase in total cell area. This may be directly ascribed to an enhancement of cell adhesion and spreading. 

The differentiation process is strongly related to the degree of cell spreading [58]. In particular, an increased cell spreading would improve the osteogenic differentiation of hMSCs due to a potential enhancement of the cytoskeletal contractility that favors osteogenesis [58].

Comparing the different cell-laden substrates at 7 and 14 days, the lower values of the cell shape factor for the magnetically-stimulated PCL/Fe_3_O_4_ nanocomposites were consistent with the results from the normalized ALP activity, justifying the differences found in the differentiation process. 

Anyway, osteogenic differentiation is a complex process that involves several biophysical cues and biological factors [59]. Even if cell spreading, which was measured through shape factor, plays a crucial role in the regulation of these process, many concerns remain on how it may influence the maintenance of the committed phenotype after MSC differentiation [59].

On the other hand, the phosphorylation levels of ERK1/2 also evidenced the role of the material–magnetic field combination. 

Specifically, the possibility to activate the MAPK pathway was demonstrated for both magnetically-stimulated and unstimulated PCL and PCL/Fe_3_O_4_ substrates in the presence of EGF (Figure 7).

The magnetic stimulation positively influenced the EGF-dependent phosphorylation of ERK and also provided interesting results in the absence of EGF, especially for PCL/Fe_3_O_4_ substrates (Figure 7). 

The application of a time-dependent magnetic field improved the activation of the MAPK pathway, as evidenced by an increase of the ERK phosphorylation for both PCL and PCL/Fe_3_O_4_.

It is also worth noting how the best results in terms of ERK phosphorylation were achieved by combining magnetic stimulation and magnetic nanocomposites (PCL/Fe_3_O_4_ Mag), thus stressing the important role of the material–magnetic field combination (Figure 7).

Accordingly, the obtained findings would seem to corroborate the role of magnetic stimulation.

Strategies to improve bone tissue regeneration, involving magnetic nanocomposite scaffolds with or without the application of a magnetic field, have been widely reported in the literature [42]. 

ALP activity, the mRNA expression of specific markers (e.g., osteocalcin and osteopontin) and alizarin red staining on nanocomposite scaffolds consisting of PCL and magnetite nanoparticles showed how magnetic features can stimulate the differentiation process [60]. The potential to upregulate specific integrin subunits and to activate downstream pathways (e.g., focal adhesion kinase-FAK, paxillin, p38, and ERK/MAPK) was reported [60].

Furthermore, runt-related transcription factor 2 (Runx2) has an important role in promoting bone regeneration, and several factors that are involved in osteogenic differentiation regulate its expression [61,62]. Runx2 phosphorylation is also mainly mediated by ERK1/2 [62].

The combination of a static magnetic field with magnetic PCL/Fe_3_O_4_ scaffolds provided interesting information in terms of ALP activity as well as of the expression of bone-associated genes (e.g., Runx2 and Osterix). The role of such a combination further evidenced the possibility to activate integrin signaling pathways (e.g., FAK, paxillin, and Ras homolog family member A-RhoA), as well as to upregulate bone morphogenetic protein-2 and the phosphorylation of Smad 1/5/8 [63].

For this reason, the current study may be considered as a first step toward a research involving the combination design of 3D additive manufactured magnetic scaffolds and a time-dependent magnetic field, with the aim to provide a prompt on the assessment of the behavior of hMSCs through further analyses on osteogenic differentiation, involving alizarin red staining and the gene expression of osteogenic markers such as bone morphogenetic protein-2 (BMP-2), Runx2, and collagen type 1 alpha 1 (COL1A1), and to study the MAPK pathway alterations related to magnetic stimulation in cell-laden constructs.

## 5. Conclusions

Within the limitations of the proposed research, the following conclusions were reached:

1. The possibility to develop PCL/Fe_3_O_4_ (80/20 w/w) substrates with improved mechanical properties (higher strength than PCL without negatively affecting the work to failure) was demonstrated by the small punch test.
2. The combination of a time-dependent magnetic field with PCL/Fe_3_O_4_ nanocomposites (PCL/Fe_3_O_4_ Mag) impacted the behavior of hMSCs, especially resulting in a prolonged cell differentiation.
3. The effect of a time-dependent magnetic field in increasing ERK phosphorylation levels and, hence, in the activation of the MAPK pathway, was reported, also corroborating previous findings on the combination of a magnetic field and magnetic nanocomposite structures.
4. The role of the material–magnetic field combination was revealed, as the highest ERK phosphorylation levels were found in the case of PCL/Fe_3_O_4_ Mag.

## Figures and Tables

**Table 1:** Results from small punch tests: peak load and work to failure.

Materials	Peak Load (N)	Work to Failure (mJ)
PCL	28.1 ± 2.1	31.4 ± 4.2
PCL/Fe_3_O_4_ (80/20 w/w)	34.4 ± 2.6	30.1 ± 5.3
